# The role of autophagy in the pathogenesis of exposure keratitis

**DOI:** 10.1111/jcmm.14310

**Published:** 2019-04-11

**Authors:** Guoliang Wang, Yuhua Xue, Yanzi Wang, Fei Dong, Mei Shen, Rongrong Zong, Zuguo Liu, Cheng Li

**Affiliations:** ^1^ Eye Institute & Affiliated Xiamen Eye Center School of Medicine, Xiamen University Xiamen China; ^2^ Fujian Provincial Key Laboratory of Ophthalmology and Visual Science Xiamen China; ^3^ School of Pharmaceutical Sciences Xiamen University Xiamen China

**Keywords:** air exposure, autophagy, corneal epithelium, endoplasmic reticulum stress, exposure keratopathy

## Abstract

Incomplete tear film spreading and eyelid closure can cause defective renewal of the ocular surface and air exposure‐induced epithelial keratopathy (EK). In this study, we characterized the role of autophagy in mediating the ocular surface changes leading to EK. Human corneal epithelial cells (HCECs) and C57BL/6 mice were employed as EK models, respectively. Transmission electron microscopy (TEM) evaluated changes in HCECs after air exposure. Each of these models was treated with either an autophagy inhibitor [chloroquine (CQ) or 3‐methyladenine (3‐MA)] or activator [Rapamycin (Rapa)]. Immunohistochemistry assessed autophagy‐related proteins, LC3 and p62 expression levels. Western blotting confirmed the expression levels of the autophagy‐related proteins [Beclin1 and mammalian target of rapamycin (mTOR)], the endoplasmic reticulum (ER) stress‐related proteins (PERK, eIF2α and CHOP) and the PI3K/Akt/mTOR signalling pathway‐related proteins. Real‐time quantitative PCR (qRT‐PCR) determined IL‐1β, IL‐6 and MMP9 gene expression levels. The TUNEL assay detected apoptotic cells. TEM identified autophagic vacuoles in both EK models. Increased LC3 puncta formation and decreased p62 immunofluorescent staining and Western blotting confirmed autophagy induction. CQ treatment increased TUNEL positive staining in HCECs, while Rapa had an opposite effect. Similarly, CQ injection enhanced air exposure‐induced apoptosis and inflammation in the mouse corneal epithelium, which was inhibited by Rapa treatment. Furthermore, the phosphorylation status of PERK and eIF2α and CHOP expression increased in both EK models indicating that ER stress‐induced autophagy promoted cell survival. Taken together, air exposure‐induced autophagy is indispensable for the maintenance of corneal epithelial physiology and cell survival.

## 1 INTRODUCTION

The outer layer of the normal cornea is composed of a non‐keratinized, non‐secretory epithelium covered with a tear film layer. It plays a pivotal role in the maintenance of normal ocular surface functions and refractive properties.^1^ Eyelid closure and blinking contribute to maintaining ocular surface integrity since they prevent the corneal surface from desiccation and compromise of epithelial barrier function, which may induce oxidative stress and deprive corneal epithelial cells of essential nutrient support.^2^

If air exposure EK develops as a consequence of defective epithelial renewal, incomplete eyelid closure and tear spreading, this pathological condition may lead to chemosis, corneal erosion, melting, infectious keratitis and even corneal perforation.^3^, ^4^ The clinical diagnosis of EK is based on patient history and physical examination findings. Patients frequently present with a presumed diagnosis of ‘dry eye’, but extended corneal air exposure is the underlying aetiology revealed by ophthalmic examination. To unravel the pathophysiology of this condition and other ocular surface diseases,^5^, ^6^ Li et al constructed an ex vivo model of air exposure keratopathy, in which human limbal explants were cultured at an air‐liquid interface (airlift).^7^

Autophagy reutilizes cellular proteins and damaged organelles to derive metabolic energy during starvation or stress.^8^, ^9^ This function plays a pivotal role in cell survival in many diseases.^10^, ^11^ Cell death can be induced in high autophagy cells by degradative processes such as ischaemia/reperfusion‐induced death of cardiac myocytes. Furthermore, exorbitant autophagy activation can also contribute to pathological changes such as the liver fibrosis and cirrhosis.^12^, ^13^ Alternatively, autophagy activation can be beneficial during various pathological and physiological states because this response protect cells from compromise of their function by recycling and degrading damaged or dysfunctional organelles, and meanwhile provides a defensive power against infection. In addition, it plays a salutary role in diabetes, heart failure and neurodegenerative diseases.^14^ It is noteworthy that, in some diseases, such as cancer, it is still not fully understood whether autophagy exerts beneficial or detrimental effects.^15^, ^16^ Thus, treatment of many human diseases can be facilitated by determining the role of autophagy in their pathophysiology.

Up to now, there is no clarity in the literature confirming the role and the extent of autophagy in the process of EK. In this study, we established both in vitro and in vivo air exposure corneal epithelial keratopathy models to determine the contributory roles of autophagy to this process. The results clarify both the role of autophagy in the survival and death of corneal epithelial cells during air exposure keratopathy and its contribution to the underlying pathophysiology.

## 2 MATERIALS AND METHODS

### 2.1 Materials and reagents

Chloroquine (CQ) and 3‐methyladenine (3‐MA) were purchased from Sigma‐Aldrich Corp. (St. Louis, MO, USA). Dulbecco's Modified Eagle Medium: Nutrient Mixture F‐12 (DMEM‐F12) media, foetal bovine serum (FBS), mouse epidermal growth factor (EGF), Alexa Fluor 488‐ and Alexa Fluor 594‐conjugated IgG were purchased from Life Technologies (Carlsbad, CA, USA). Rapamycin (Rapa), the antibodies of anti‐LC3B (ab63817), anti‐Beclin1 (ab55878) and anti‐SQSTM 1/p62 (ab56416) were purchased from Abcam (Cambridge, CS, UK). The antibodies specific for PERK (3192), phospho‐PERK (Thr980, 3179), mTOR (2983), phospho‐mTOR (Sre2448, 2971), AKT (4691), phospho‐AKT (Sre473, 9271), phosphor‐ULK1 (Ser555, 5869) and CHOP (2895) were purchased from Cell Signaling Technology (Boston, MA, USA). Salubrinal (Sal) was purchased from Amquar Bio Corp. (Denver, CO, USA). Anti‐β‐actin antibody was purchased from Santa Cruz Biotechnology (Dallas, TX, USA). Inserts used in this study were purchased from Merck Millipore (Bedford, MA, USA). Plastic cell culture dishes and six‐well plates were purchased from BD Biosciences (Lincoln Park, NJ, USA).

### 2.2 In Vivo experimental procedures

C57BL/6 mice (female, 20‐22 g) were purchased from Shanghai Shilaike Laboratory Animal Co., Ltd. (Shanghai, China). All animal experiments conducted in this study were carried out in strict accordance with the Association for Research in Vision and Ophthalmology (ARVO) Statement for the Use of Animals in Ophthalmic and Vision Research. The experimental protocol was approved by the Animal Ethics Committee of Xiamen University School of Medicine (approval ID: XMUMC: 2016‐08‐10). The mice were kept in individually ventilated cages, with food and water ad libitum.

The mouse air exposure keratopathy model procedure was performed, as described previously, with some modifications for mice.^17^ All procedures with animals were performed under general anaesthesia induced by intraperitoneal injection of ketamine hydrochloride (100 mg/kg) and xylazine hydrochloride (12.5 mg/kg). Eye speculums for mice were used to prevent eyelid closure for either 30 min, 1 h, 2 h or 4 h). Experimental mice were killed 24 hours after the initial exposure, and the corneas were collected for qRT‐PCR. The other corneal samples were immediately collected from the mice after exposure treatment.

### 2.3 Cell culture

HCECs, simian virus 40 transformed, were obtained from RIKEN Biosource Center, Tokyo, Japan, and were passaged in DMEM‐F12 supplemented with 6% heat‐inactivated FBS, bovine insulin (7 μg/mL), human epidermal growth factor (7 ng/mL), and 1% penicillin and streptomycin. For cell airlift cultures, the HCECs were plated at a density of 1 × 10^5^ cells/cm^2^ into type I collagen‐coated six‐well inserts. When the cultured HCECs reached 80% confluency, the medium was replaced with 0.6 mL fresh medium to keep the cells at the air‐liquid interface. In some airlift cultures, specific autophagy inhibitors, either CQ (10 μM) or 3‐MA (5 mM) was added to the culture medium. Cells were cultured at 37 ℃ in 5% CO_2_ and the medium was replaced every 2 days.

### 2.4 Slit‐lamp microscopic observation

Mice were examined under the slit lamp microscope 24 hours after the air exposure treatment. All of the corneal images were taken by an experienced researcher. The corneal epithelial barrier function was detected under cobalt blue light with 0.5% fluorescein sodium eye drops.

### 2.5 Transmission electron microscopy (TEM)

Cultured cells or corneas were collected and immediately fixed in 2.5% glutaraldehyde in 0.2 M phosphate buffer saline (PBS, pH 7.2) followed by 2% aqueous osmium tetroxide. Subsequently, they were dehydrated using graded ethanol series and then embedded. Semithin (0.5 μm) sections were collected to assess sample quality under a light microscope. Ultrathin (70 nm) sections were collected on copper grids and stained with uranyl acetate and lead citrate, then analysed in a transmission electron microscope system (JEM 1010; JEOL, Tokyo, Japan).

### 2.6 RNA isolation and quantitative real‐time PCR (qRT‐PCR)

The total cellular or corneal RNA was extracted using TRIzol reagent (Invitrogen, Carlsbad, CA, USA), according to the manufacturer's instruction. RNA sample parameters and concentrations were determined with a NanoDrop 1000TM spectrophotometer (Thermo Fisher Scientific, Waltham, MA, USA). The ExScript RT Reagent kit (Takara Bio, Otsu, Shiga, Japan) was used to synthesize cDNA by using 10 ng of total RNA as template. With a SYBR Premix Ex Taq Kit (Takara Bio, Otsu, Shiga, Japan) and the StepOne Real‐Time PCR detection system (Applied Biosystems, Darmstadt, Germany), qRT‐PCR was performed. The amplification program was carried out as described previously.^18^ The results of the relative qRT‐PCR were normalized to the geometric mean of the housekeeping gene, β‐actin. Relative expression levels were calculated by the 2^‐ΔΔCt^ method, where ΔΔC_t_ = (C_t, target_−C_t, β‐actin_)_sample_−(C_t, target_−C_t, β‐actin_)_normal_ (Ct represents the threshold cycle for each transcript). The primers used to amplify specific gene products from cDNA included: IL‐1β, forward primer 5′‐GAAGAAGAGCCCATCCTCTG‐3′ and reverse primer 5′‐TCATCTCGGAGCCTGTAGTG‐3′; IL‐6, forward primer 5′‐AGTCCGGAGAGGAGACTTCA‐3′ and reverse primer 5′‐TTGCCATTGCACAACTCTTT‐3′; MMP9, forward primer 5′‐TCCTTGCAATGTGGATGTTT‐3′ and reverse primer 5′‐ CTTCCAGTACCAACCGTCCT‐3′; β‐actin, forward primer 5′‐GAGACCTTCAACACCCCAGC‐3′ and reverse primer 5′‐ATGTCACGCACGATTTCCC‐3′.

### 2.7 Histological characteristics and immunostaining

Cultured HCECs or mouse corneal frozen sections were hydrated in PBS after fixed in 4% paraformaldehyde for 20 minutes, followed by incubation in 0.2% Triton X‐100 for 10 minutes. After rinsing three times with PBS for 5 minutes each and pre‐incubation for 1 hour with 2% bovine serum albumin at room temperature to block non‐specific staining, they were incubated with primary antibodies (anti‐LC3B and SQSTM1/p62) overnight at 4℃. After washing with PBS three times for 10 minutes each, specimens or cells were incubated with secondary antibodies for 1 hour. After washing with PBS three additional times for 15 minutes, sections or cells were counterstained with 4′,6‐diamidino‐2‐phenylindole (DAPI) (Dalian Meilun Biotechnology Co., Ltd, Dalian, China) and then mounted for analysis under a Leica DM2500 microscope (Leica Microsystems, Wetzlar, Germany).

### 2.8 Western blot assay

Cells and corneas were harvested in RIPA buffer (Cell Signaling Technology, Boston, MA, USA). Then they were lysed on ice for 30 minutes, before being centrifuged at 4°C at 14,000 rpm for 10 minutes. After removal of the precipitate, the concentration of extracted proteins was determined with the BCA assay using a commercial kit (Thermo Fisher Scientific, Waltham, MA, USA). Then, the protein samples adjusted to the same concentration were mixed thoroughly with 5 × SDS loading buffer, and heated for 10 minutes at 100°C for denaturation. The protein samples were separated by electrophoresis on 8% or 12% SDS‐PAGE gels, and then transferred onto polyvinylidene difluoride membranes (Roche, Indianapolis, IN, USA). After being blocked in 2% BSA for 1 hour, the membranes were incubated with a primary antibody (1:1000) overnight at 4°C. Primary antibodies against LC3B, SQSTM1/p62, Beclin1, CHOP, AKT, p‐AKT, mTOR, p‐mTOR, PERK and p‐PERK were used in this study. On the next day, membranes were rinsed in TBST for 10 minutes thrice, and then incubated with horseradish peroxidase‐conjugated goat anti‐rabbit or anti‐mouse IgG (Bio‐Rad, Hercules, CA, USA) (1:10000) for 1 hour. After three rinsed in TBST, a membrane was analysed in a ChemiDoc XRS imaging system (Bio‐Rad). The optical density (OD) was determined using the software of Quantity One. Anti‐β‐actin mouse monoclonal antibody (1:10000) acted as a loading control.

### 2.9 In Situ TUNEL assay

To measure end‐stage apoptosis, in situ terminal deoxynucleotidyl transferase dUTP nick end labelling (TUNEL) was performed on the corneas or HCECs after air exposure for various durations, according to the DeadEnd Fluorometric TUNEL System protocol. Cells or sections were counterstained in mounting medium with DAPI after TUNEL. The fluorescent dye‐conjugated dUTP‐labelled DNA and DAPI were visualized under a confocal laser scanning microscope (Fluoview FV1000; Olympus, Tokyo, Japan).

### 2.10 Statistical analysis

We conducted a one‐way ANOVA test to analyse the cell counting data, Western blot and quantitative real‐time PCR, followed by a post hoc analysis Tukey test or a Student's *t* test established significance of differences between the groups. A value of *P* < 0.05 was considered statistically significant.

## 3 RESULTS

### 3.1 Autophagy activation in air‐exposed human corneal epithelial cells

**Figure 1:**
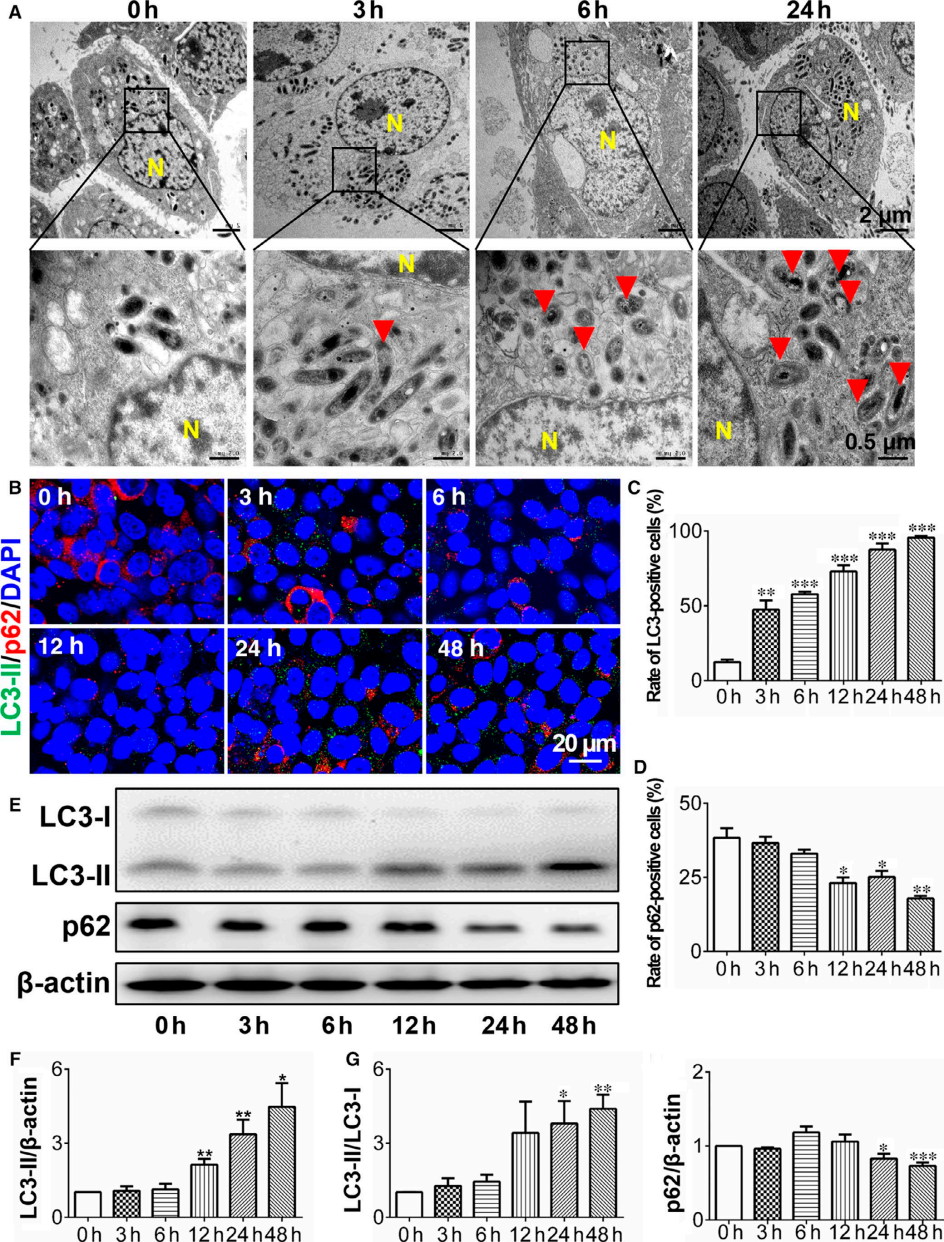
Increased autophagy in HCECs after air exposure treatment. A, Air‐lifted HCECs were subjected to this condition for 0, 3, 6 and 24 h, and then subjected to TEM analysis. The autophagic vacuoles are indicated by red triangles. Nuclei are designated by the letter N. Representative images are shown from three independent experiments. B, Immunofluorescent staining showed the changes of LC3‐II and p62 expression in the HCECs treated with different exposure times (0 h, 3 h, 6 h, 12 h, 24 h and 48 h). The LC3‐II puncta were significantly increased, while p62 aggregation number were decreased with air exposure treatment, n = 3. C, D, Quantification of the LC3‐II and p62 punctum percentage in A. E, Western blots with anti‐LC3 and p62 antibodies after air exposure treatment, n = 3. F, G, H, Quantification of the Western blot in (D). *P < 0.05, **P < 0.01, ***P < 0.001

Only scant reports described the details of air exposure‐induced autophagy in ocular tissues. The HCECs were cultured under an air‐lift condition for 3, 6 and 24 hours. The formation of autophagosomes was observed in our study by TEM, which is one of the most important signs of autophagic activity. Then they fuse with lysosomes to form single‐membrane bound degradative vacuoles (autolysosomes). Double‐membraned autophagosomes and autolysosomes are evident in TEM micrographs of air‐lifted HCECs at 3, 6 and 24 hours show existing cells. However, no such structures were present in the control submerged group. Moreover, at 24 hours more autophagic vacuoles were present than at 3 hours, which is indicative of a time‐dependent process activated by air‐lift HCEC cultures (Figure 1A).

To verify the TEM results, immunofluorescent staining and Western blotting analysis were performed to determine if increases occurred in the expression levels of the autophagy‐related proteins, microtubule‐associated protein 1 light chain 3 (LC3) 19 and sequestosome 1 (SQSTM1/p62, p62).^20^ When autophagy occurs, punctate LC3 protein expression becomes evident. Furthermore, the soluble form of LC3 (LC3‐I) is transferred to ATG3 and converted into the lipidated and autophagosome‐associated form (LC3‐II). p62 is a specific substrate that interacts with ubiquitin and triggers the degradation of proteins in the proteasome or lysosome. In our study, immunofluorescent staining showed that the proportion of LC3‐II‐positive cells increased, whereas that of p62‐positive cells decreased after air‐lift exposure treatment (Figure 1B‐D) (*P* < 0.05). Western blotting showed that the ratio of LC3‐II/LC3‐I was significantly higher in air‐lifted cells than that in untreated controls, while the p62 protein level was reduced 24 hours after the air‐lift period (Figure 1E‐H). These findings demonstrate that the air‐lift method effectively induces autophagy in HCECs in a time‐dependent manner.

### 3.2 Autophagy improves air‐lifted HCECs survival

**Figure 2:**
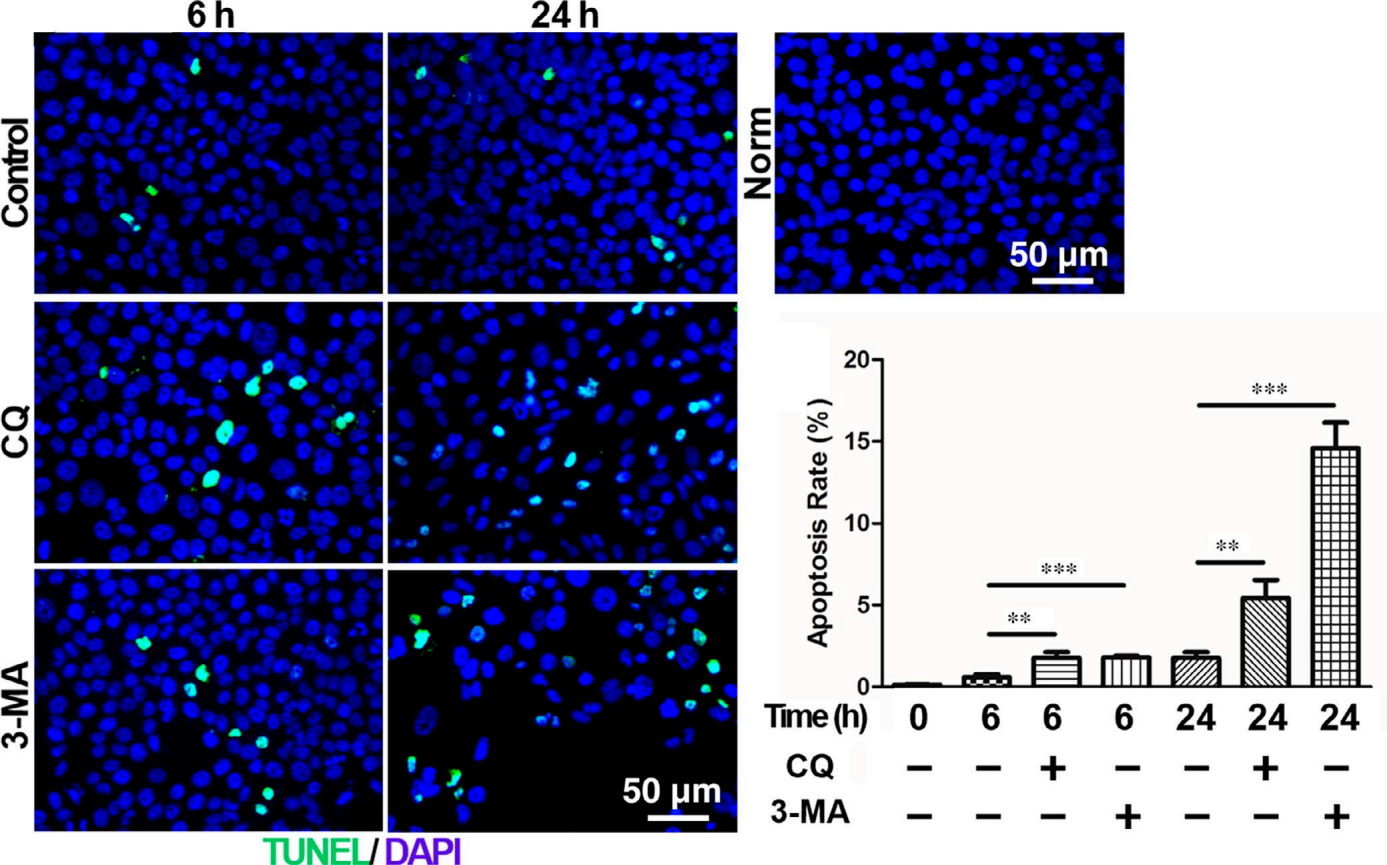
Autophagy modulates the apoptosis of HCECs after air exposure. A, Immunofluorescence staining with TUNEL in the HCECs after different treatments, n = 3. B, Quantification of the percentage of TUNEL positive cells. The proportions of TUNEL positive cells in the 6 h and 24 h air‐lifted cultured groups were significantly greater than the undamaged controls. Moreover, the numbers of apoptotic cells were significantly increased by CQ and 3‐MA, n = 3. **P < 0.01, ***P < 0.001

Autophagy may have different effects on cell survival in different environments.^21^ To make such an assessment in HCECs, the individual effects of the autophagy inhibitors, [chloroquine (CQ; 10 μM) or 3‐methyladenine (3‐MA; 5 mM)] on apoptosis was evaluated in air‐lifted HCECs. TUNEL staining was conducted to observe the effect of autophagy on the survival of air‐lifted HCECs. In medium submerged HCECs, almost no apoptosis was seen (Figure 2A); however, in an air‐lifted HCECs culture, in the absence of an autophagy inhibitor, apoptosis was evident, although no significant difference was seen between 6 and 24 hours. Compared with the normal control group, in the presence of the autophagy inhibitors, CQ or 3‐MA, the number of apoptotic cells increased which was accompanied by a significant decline in the total cell number (Figure 2B).

### 3.3 An air exposure‐induced corneal injury mouse model causes autophagy

**Figure 3:**
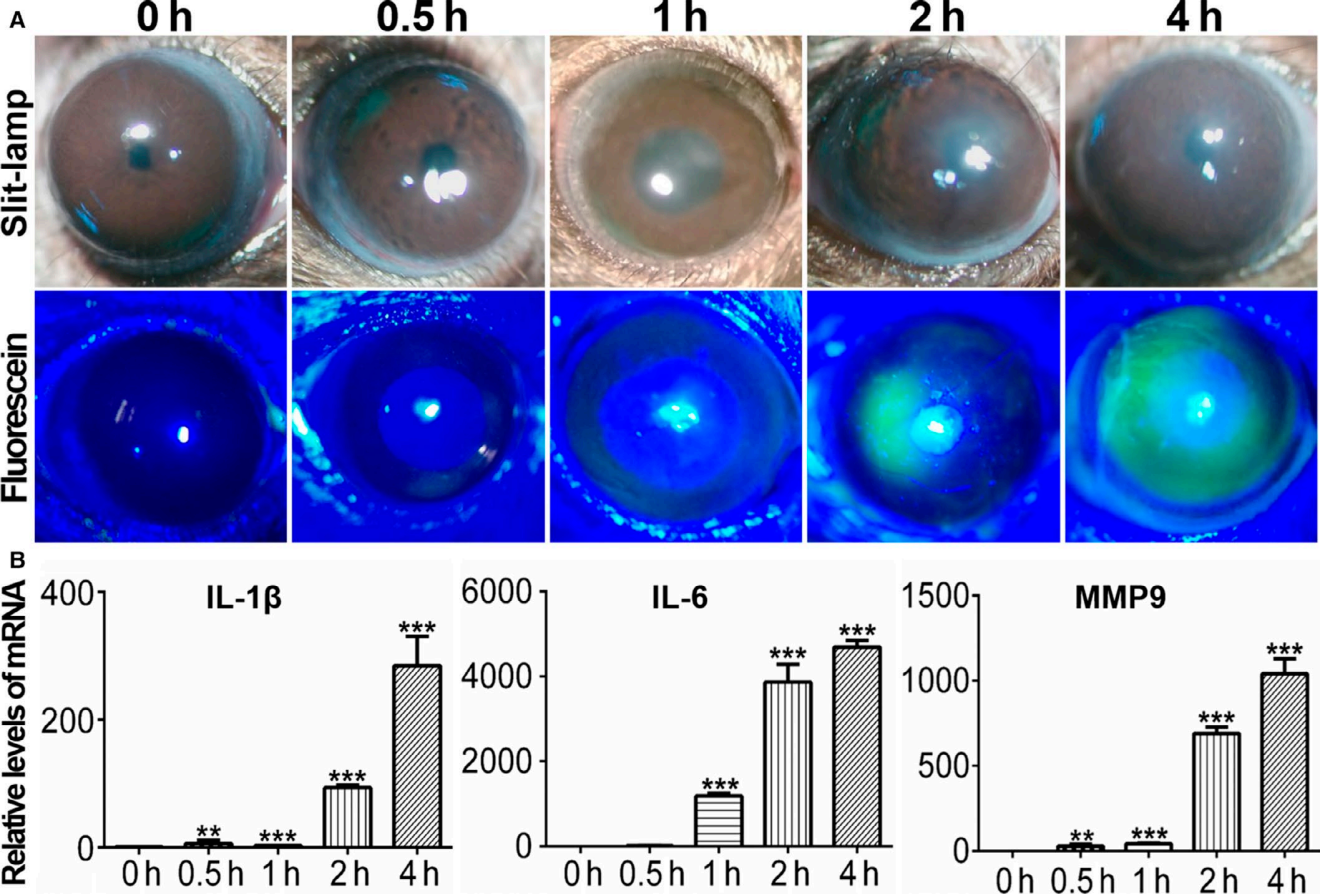
Ocular surface injury and expression of inflammation‐related factors after corneal air‐lift treatment. A, Slit‐lamp microscopy revealed a gradual increase of corneal oedema and fluorescein scans in the cornea treated with different exposure times (0h, 0.5 h, 1 h, 2 h and 4 h), n = 3. B, The mRNA levels of IL‐1β, IL6 and MMP9 were analysed by qRT‐PCR after different exposure times, n = 3. **P < 0.01, ***P < 0.001

To determine the effect of air‐lifting on autophagy induction in vivo, we developed an air exposure injury mouse model.^17^ During time‐dependent increases in air‐lift exposure time lasting up to 24 hours, corneal transparency and fluorescein sodium staining (Figure 3A) were not significantly different in the 0.5 hours exposure group from those in the control group. On the other hand, in the 1, 2 and 4 hours exposure groups, corneal turbidity progressively developed along with extensive fluorescein sodium staining (Figure 3A), which was accompanied by corneal inflammation and conjunctival hyperaemia. In addition, qRT‐PCR revealed that in air‐lifted corneas, the proinflammatory factors IL‐1β, IL6 and degradative MMP9 were significantly elevated in a time‐dependent manner (Figure 3B).

**Figure 4:**
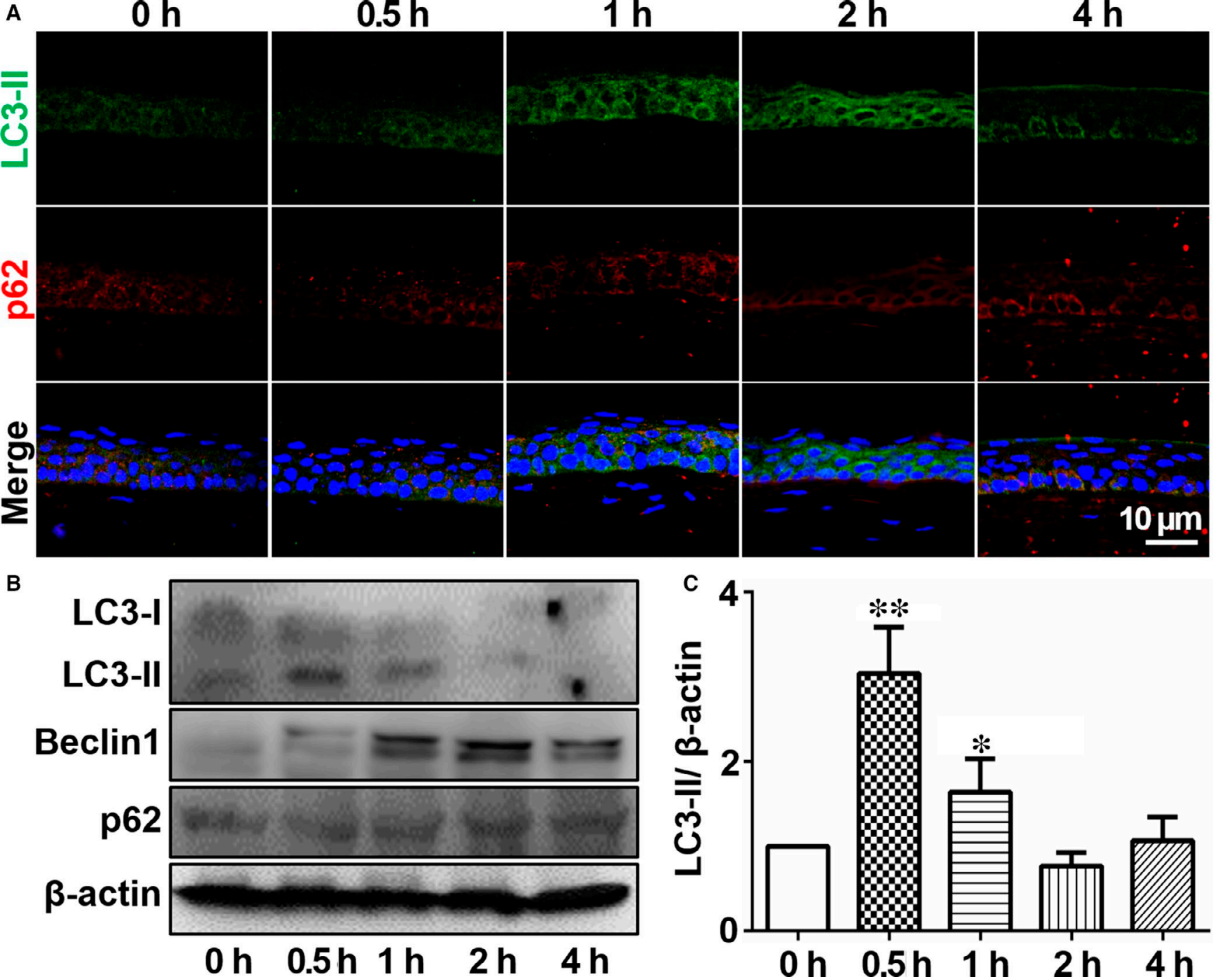
Increased autophagy in mouse corneal epithelium after air‐lifted treatment. A, Immunofluorescence staining with the anti‐LC3‐II and p62 antibodies in the mouse corneal epithelium after different exposure times (0 h, 0.5 h, 1 h, 2 h and 4 h), n = 3. B, Western blot showing the changes in LC3‐II, Beclin1 and p62 expression in the corneal epithelium after air exposure treatment, n = 3. C, Quantification of the western blot result for LC3‐II in (B). *P < 0.05, **P < 0.01

Immunofluorescent and Western blotting analysis were performed to verify autophagy activation in the corneal air exposure injury mouse model. LC3 was expressed was mostly scattered with a few spots in the cytoplasm of normal mouse corneas (Figure 4A), indicating possible basal autophagy. Following air‐lifted treatment, the fluorescence intensity of LC3 gradually increased while that of p62 diminished, indicating that the air‐lifted treatment may induce epithelial cell autophagy. However, after 4 hours of air‐lifted corneal exposure, the LC3 fluorescence intensity decreased suggesting that autophagy may begin to wane after a 2 hours exposure. Western blot analysis showed that LC3II expression increased at 0.5 hours, and Beclin1 was significantly up‐regulated at 1 hour (Figure 4B and C). These results suggest that autophagy was activated in this air‐lifted autophagy mouse cornea model.

### 3.4 Autophagy improves corneal epithelial cell survival in an air‐lifted mouse autophagy model

**Figure 5:**
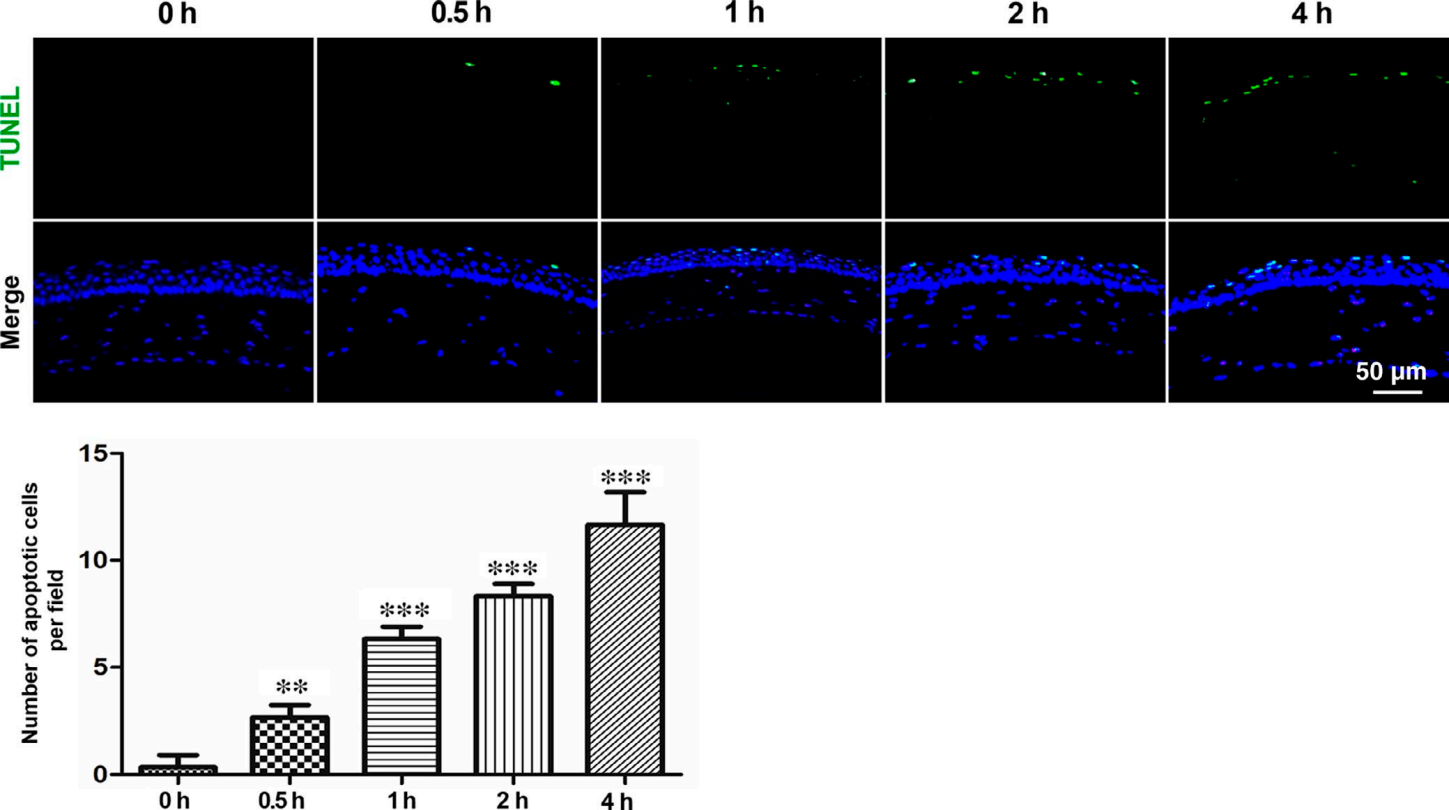
Increased apoptotic cells in mouse corneal epithelium after air exposure treatment. A, Images of TUNEL staining in the mouse cornea after different air exposure times, n = 3. B, Quantification of the number of TUNEL positive cells in (B), **P < 0.01, ***P < 0.001

The aforementioned results suggest that autophagy activation protects air‐lifted HCECs from apoptosis (Figure 2); and interestingly, basal autophagy developed in the air‐lifted mouse model (Figure 4). To verify this protective effect of autophagy on in vivo corneal epithelial cells, the TUNEL assay was performed. Compared with the control group, the number of apoptotic corneal epithelial cells increased significantly with the extended exposure time (Figure 5A). The differences were statistically significant amongst each of the groups (Figure 5B).

**Figure 6:**
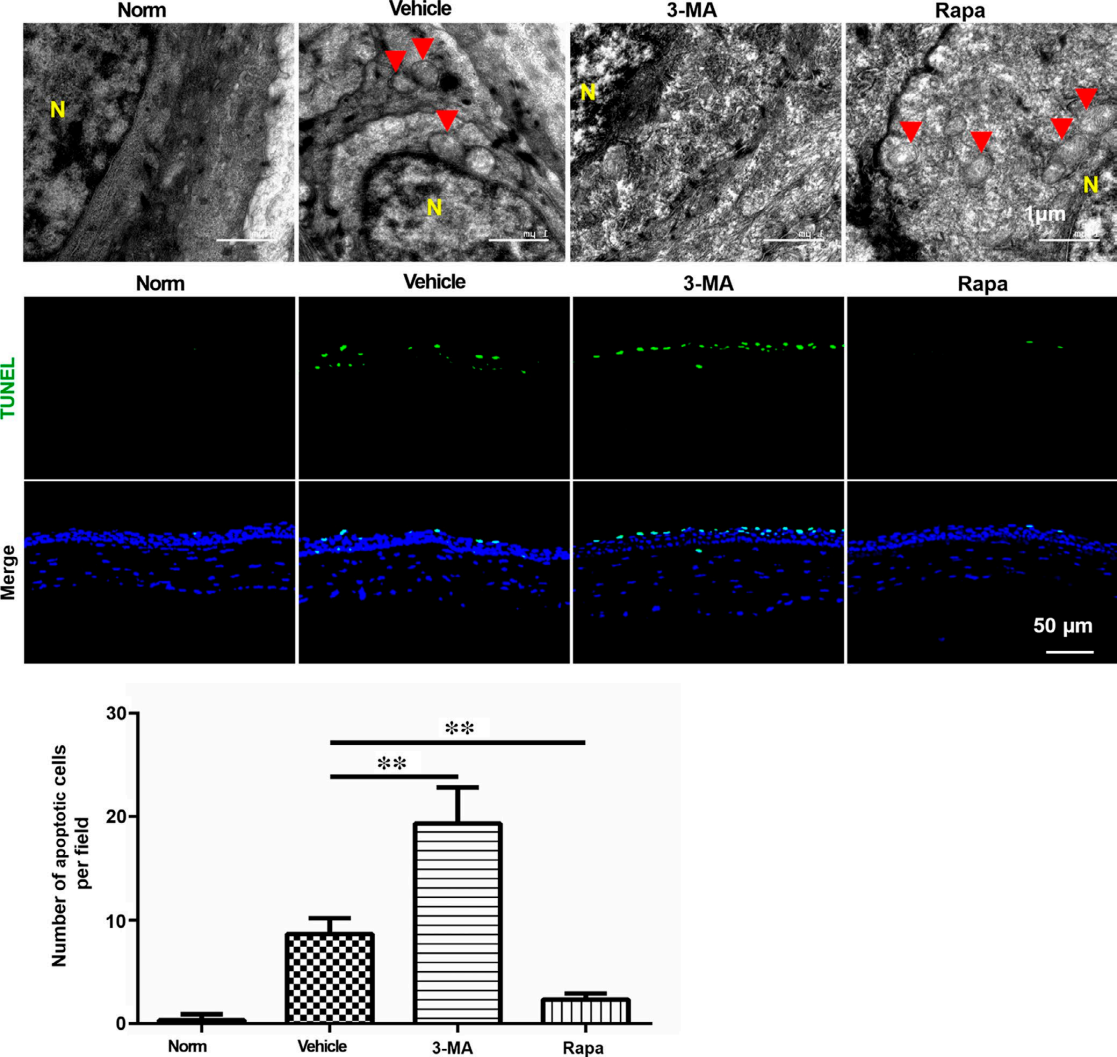
Rapamycin and 3‐MA affected both the induction of autophagy and survival in the corneal epithelium after air exposure damage. A, TEM analysis to evaluate the presence of autophagy in mouse corneal epithelium. Norm: normal control group, Vehicle: solvent injection group, 3‐MA: 3‐MA injection group, Rapa: rapamycin injection group. Autophagosomes are indicated by the red triangle. Nuclei are designated by the letter N, n = 3. B, Immunofluorescence staining with TUNEL in the corneal epithelium after different treatments, C, Quantification of the number of TUNEL positive cells in (B), The number of TUNEL positive cells in Vehicle group was significantly greater than the untreated control. Moreover, the numbers of apoptotic cells were significantly increased by 3‐MA and decreased by rapamycin, n = 3. **P < 0.01

To evaluate the individual effects of autophagic activation and inhibition on this process, a novel corneal exposure injury mouse model was subsequently implemented with intraperitoneal injection of Rapa (15 mg/kg), an autophagic activator, and 3‐MA (15 mg/kg), an autophagic inhibitor for 2 hours prior to performing corneal air‐lift for additional 2 hours. Rapa is a commonly used reagent that induces autophagy by inhibiting the Ser/Thr protein kinase mTOR. On the other hand, 3‐MA inhibits autophagy by blocking the autophagosome formation via inhibition of class III PI3K. The TEM images showed autophagic vacuoles in the mouse corneal epithelium in the solvent vehicle group, and Rapa pre‐treatment caused the number of autophagic vacuoles to slightly develop. In contrast, no autophagic vacuoles were identifiable in the 3‐MA group, indicating that 3‐MA effectively inhibited the activation of autophagy in the corneal epithelial cells in this mouse autophagy air‐lifted corneal epithelial model (Figure 6A). Based on these results, the eyes were then isolated in preparation for TUNEL staining. There was no obvious difference in staining between the CQ and solvent groups; however, cell apoptosis increased in the 3‐MA group but it decreased in the Rapa group as compared with their solvent vehicle groups (Figure 6B). Statistical analysis showed that the differences of the number of apoptotic cells between CQ or 3‐MA treated group and solvent group were statistically significant (Figure 6C). In conclusion, Rapa injection activated autophagy and boosted the survival of epithelial cells in this air‐lifted autophagy mouse model.

### 3.5 Air exposure induces endoplasmic reticulum (ER) stress in corneal epithelial cells and activates autophagy via the PI3K/AKT/mTOR signalling pathway

**Figure 7:**
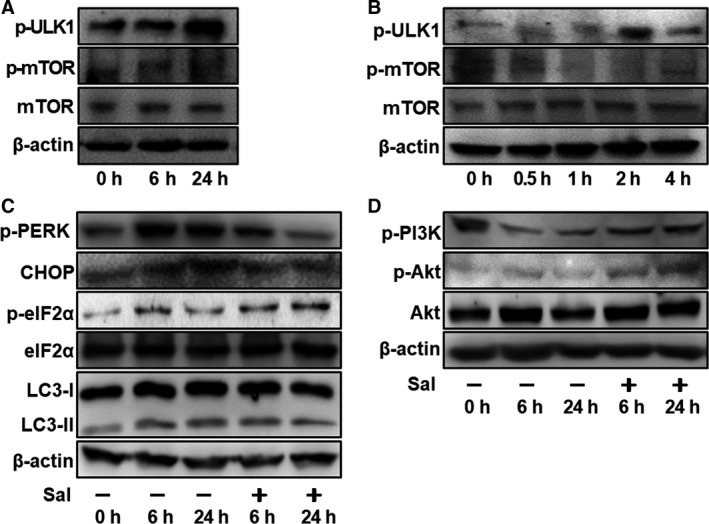
Autophagy induced by air‐lift injury is mTOR‐dependent, and is regulated by ER stress via the PI3K/Akt/mTOR pathway. A, Western blot showing the changes in p‐ULK1, p‐mTOR and mTOR expression in the HCECs cells treated with different air exposure times (6 h and 24 h), n = 3. B, Western blots with anti‐ p‐ULK1, p‐mTOR and mTOR antibodies in air‐lifted corneal epithelium with different exposure times (0 h, 0.5 h, 1 h, 2 h and 24 h), n = 3. (C, D) HCECs were incubated with DMSO or 10 μM salubrinal (Sal) for 6 h, and followed by air exposure for 6 h or 24 h. C, Western blot analysis shows that sal could restore air exposure increased p‐PERK, CHOP and LC3‐II and decreased p‐eIF2α, n = 3. D, Sal also restores air exposure‐induced phosphorylation of PI3K and Akt, n = 3

Autophagy (macroautophagy) is induced through both the mechanistic target of rapamycin (mTOR)‐dependent autophagy and non‐mTOR‐dependent autophagy pathways. mTOR regulates the autophagic process by modifying the phosphorylation of Unc‐51‐like autophagy activating kinase 1 (ULK1), while the activation of mTOR is controlled by the upstream protein kinase B (AKT).^22^ To determine the mTOR involvement in air‐lift induced autophagy, the individual effects were examined of this stress on mTOR and ULK1 expression levels. The results indicate that in this model the mTOR phosphorylation status was inhibited since the p‐mTOR content decreased, which is consistent with the increased p‐ULK1 content (Figure 7A and B). These effects were consistent with those in the in vitro autophagy HCECs model suggesting a commonality in upstream signalling control of this process.

The mTOR phosphorylation status is regulated by endoplasmic reticulum (ER) stress in some tissues. In these studies, it was shown that protein kinase R‐like endoplasmic reticulum kinase (PERK), CCAAT‐enhancer binding protein homologous protein (CHOP) and eukaryotic initiation factor 2α (eIF2α) play important roles in modulating control of mTOR phosphorylation. Therefore, changes in their expression levels serve as markers of mTOR phosphorylation involvement.^23^ Similarly, PERK, CHOP and eIF2α in HCECs can serve as readouts of this control and Western blotting showed that they actually increased in a time‐dependent manner. At 6 and 24 hours after imposing the air‐lift condition, autophagy increased in HCECs as indicated by an increase in LC3 expression and the content of ER stress‐related proteins, phosphorylated PERK (p‐PERK), CHOP and phosphorylated eIF2α (p‐eIF2α) also increased significantly (Figure 7C). In contrast, following addition of the ER stress inhibitor, salubrinal (sal), the levels of p‐PERK and CHOP decreased in conjunction with the level of autophagy, as indicated by a decrease in LC3II expression. These declines occurred since sal inhibited dephosphorylation of eIF2α, which in turn increased levels of p‐eIF2α.

It is known that mTOR is involved in the regulation of air‐lift injury‐induced autophagy (Figure 7A and B). mTOR is also an important downstream kinase of PI3K/AKT, and together this signalling axis contributes to the regulation of cell proliferation and apoptosis. 24 In the present study, the air exposure condition (6 and 24 h) down‐regulated the levels of p‐mTOR in conjunction with the levels of p‐AKT and p‐PI3K. However, following the addition of sal to inhibit ER stress, phosphorylation of AKT and PI3K increased (Figure 7D). Together, these results imply that air exposure regulates the PI3K/AKT/mTOR signalling pathway via the induction of ER stress, in turn promoting protective autophagy.

## 4 DISCUSSION

Maintenance of the tear film composition and osmolality is essential for preserving corneal and conjunctival surface health. This requirement is evident since either a change in tear fluid composition or an increase in tear film osmolality may result in various ocular surface diseases.^25^, ^26^ Changes in osmotic pressure may, disrupt cellular homoeostasis, and lead to autophagy. Such an association exists in rat spinal cord cells because Jiang et al found that high glucose‐induced hyperosmolarity, inhibited 70 kDa ribosomal S6 kinase (p70S6K) expression whereas the Ca^2+^‐dependent AMPK/mTOR pathway was activated, triggering autophagy.^27^ Han et al also induced hyperosmolarity in cultured Chinese hamster ovarian cells through NaCl supplementation and described time‐dependent increases in GFP‐LC3‐positive immunostaining indicating autophagy initiation.^28^ EK caused by incomplete blink and prolonged inter‐blink intervals is associated with increased tear evaporation and hyperosmolarity. 29 In the present study, we showed that an airlift rapidly activates autophagy in an animal model and in HCECs, suggesting autophagy is an adaptive response to tear film hyperosmolarity.

Autophagy occurs as the result of activation of a degradative pathway regulating both cell survival and death. It has a dual role in disease because autophagy can be either protective or destructive to cells, which depends on treatment conditions and individual genetic signatures. Piras et al found that blocking autophagy significantly inhibited apoptosis of retinal ganglion cells (RGCs) in the rat acute glaucoma model.^30^ In contrast, Rodriguez‐Muela et al reported that autophagy inhibited RGCs apoptosis, using *Atg4B* ^‐/‐^ and *Atg5* ^‐/‐^ mice.^31^ Regarding an association between medium composition and ocular surface health, lacritin, an endogenous tear glycoprotein, reduced oxidative damage to corneal epithelial cells and maintained cellular homoeostasis by inducing autophagy.^32^, ^33^ In the present study, we also found that autophagy was rapidly induced subsequent to air‐lifted HCECs. Furthermore, following addition of either CQ or 3‐MA, autophagy inhibitors, the number of apoptotic cells rapidly increased. These results suggest that in an air‐lift model, autophagy promotes the survival of corneal epithelial cells whereas apoptosis is inhibited.

In a clinical setting, the causes of air exposure‐induced ocular surface injuries are complex. Under this condition, therapeutic management includes: (a) restoring as much as possible normal tear film composition and osmolality to promote healing of the injured ocular surface epithelium and protect the patient's visual function; (b) inhibiting ocular inflammatory responses and preventing formation of permanent lesions and other serious complications of ocular surface epithelium; (c) improving patient comfort by providing symptomatic relief.^34^ However, existing therapeutic interventions are inadequate for achieving these objectives in critically ill patients. The present study found that autophagy may be a potential therapeutic target for air‐exposed lesions, proposing a novel avenue for the treatment of ocular surface diseases.

The regulation of autophagy in the treatment of diseases has already achieved good results in various animal models. For instance, the activation of mTOR‐dependent autophagy may delay neurodegenerative symptoms in fly and mouse models of Huntington's disease,^35^ and a mouse model of Alzheimer's disease.^36^ The present study found that with increased exposure time, the levels of p‐mTOR gradually decreased, which was negatively correlated with changes in p‐ULK1 and LC3II. Therefore, this type of autophagy is considered to be mTOR‐dependent. Previously, Rapa has been used as an anti‐inflammatory drug to treat ocular surface diseases. Shah et al found that Rapa had a significant anti‐inflammatory effect in a Sjögren's syndrome mouse model, which inhibited lacrimal gland inflammation and improved ocular surface conditions.^37^ Nevertheless, Rapa is also a potent activator of mTOR‐dependent autophagy.^38^ In our mouse model, following injection of the autophagy activator, Rapa significantly decreased the number of apoptotic cells (Figure 6) which likely suggests that Rapa promotes ocular surface healing through affecting autophagy in this model.

In recent years, numerous studies have shown that ER stress induces autophagy. Gao et al described ER stress‐induced autophagy in a rat cardiac ischaemia‐reperfusion model,^39^ and Bernales et al reported autophagy involvement in the maintenance of homoeostasis in cells that developed ER stress.^40^ Consistently, we found that ER stress appears to be involved in the air‐lift model because the ER stress‐related proteins, p‐PERK, CHOP and p‐eIF2α, underwent up‐regulation. The autophagy observed in the present study was mTOR‐dependent (Figure 7A and B). We speculated that the air lift injury‐induced ER stress may regulate the initiation of autophagy via the PI3K/AKT/mTOR signalling pathway, since it is an active player in the regulation of cell proliferation and apoptosis.^24^ Our hypothesis is supported by our result showing that the ER stress inhibitor, sal (Figure 7D) activated this pathway. This result is consistent with data reported by Feng and Qin, suggesting that ER stress affects autophagy through the PI3K/AKT/mTOR signalling pathway.^41^, ^42^ Other studies showed that glaucoma medications sustained activation of ER stress in corneal epithelial cells.^43^ Okumura et al reported abnormal activation of ER stress in corneal endothelial cells isolated from patients with Fuchs' endothelial corneal dystrophy.^44^ For the first time, our study suggests that there is a link between ER stress and the initiation of autophagy in ocular surface diseases.

The multiple actions of ER stress in controlling gene expression serve as a double‐edged sword for normal development and physiology.^45^ Under pathological conditions, ER stress is initially an adaptive response, which can lead to interference with developmental signals and disruption of gene expression patterns if stress remains unresolved. Subsequently, the cell can display altered proliferation and differentiation, becoming dysfunctional. Under extreme stress conditions, an apoptotic signal is triggered, which becomes dominant and leads to cell death.^45^ In the present study, air exposure‐induced ER stress may have activated autophagy through the PI3K/AKT/mTOR pathway, which plays a positive role in the maintenance of ER homoeostasis and cell survival by degrading and recycling misfolded proteins and damaged organelles. The realization of developing medications that regulate ER stress and autophagy will improve the treatment of ocular surface diseases.

## CONFLICT OF INTEREST

The authors declare that there is no conflict of interest.
